# Rapid Environmental Change Drives Increased Land Use by an Arctic Marine Predator

**DOI:** 10.1371/journal.pone.0155932

**Published:** 2016-06-01

**Authors:** Todd C. Atwood, Elizabeth Peacock, Melissa A. McKinney, Kate Lillie, Ryan Wilson, David C. Douglas, Susanne Miller, Pat Terletzky

**Affiliations:** 1 U.S. Geological Survey, Alaska Science Center, 4210 University Drive, Anchorage, AK, 99508, United States of America; 2 University of Connecticut, Center for Environmental Sciences and Engineering and Department of Natural Resources and the Environment, 1376 Storrs Road, Storrs, CT, 06269, United States of America; 3 Utah State University, Department of Wildland Resources, Logan, UT, 84322–5230, United States of America; 4 U.S. Fish and Wildlife Service, Marine Mammals Management, 1011 E Tudor Road, Anchorage, AK, 99503, United States of America; 5 U.S. Geological Survey, Alaska Science Center, 250 Egan Drive, Juneau, AK, 99801, United States of America; Institute of Deep-sea Science and Engineering, Chinese Academy of Sciences, CHINA

## Abstract

In the Arctic Ocean’s southern Beaufort Sea (SB), the length of the sea ice melt season (i.e., period between the onset of sea ice break-up in summer and freeze-up in fall) has increased substantially since the late 1990s. Historically, polar bears (*Ursus maritimus*) of the SB have mostly remained on the sea ice year-round (except for those that came ashore to den), but recent changes in the extent and phenology of sea ice habitat have coincided with evidence that use of terrestrial habitat is increasing. We characterized the spatial behavior of polar bears spending summer and fall on land along Alaska’s north coast to better understand the nexus between rapid environmental change and increased use of terrestrial habitat. We found that the percentage of radiocollared adult females from the SB subpopulation coming ashore has tripled over 15 years. Moreover, we detected trends of earlier arrival on shore, increased length of stay, and later departure back to sea ice, all of which were related to declines in the availability of sea ice habitat over the continental shelf and changes to sea ice phenology. Since the late 1990s, the mean duration of the open-water season in the SB increased by 36 days, and the mean length of stay on shore increased by 31 days. While on shore, the distribution of polar bears was influenced by the availability of scavenge subsidies in the form of subsistence-harvested bowhead whale (*Balaena mysticetus*) remains aggregated at sites along the coast. The declining spatio-temporal availability of sea ice habitat and increased availability of human-provisioned resources are likely to result in increased use of land. Increased residency on land is cause for concern given that, while there, bears may be exposed to a greater array of risk factors including those associated with increased human activities.

## Introduction

The long-term persistence of polar bears (*Ursus maritimus*) is linked to the health of the Arctic marine ecosystem, particularly the availability of sea-ice habitat [1, 2]. Polar bears are specialist carnivores that rely on sea ice to meet a number of life history needs including accessing prey, searching for mates, and establishing maternal dens [3]. However, the Arctic region is experiencing a warming trend that is driving pronounced changes in sea ice extent and structure. Since 1979, sea ice extent and volume during summer have declined at rates of ≈14%/ and 28%/decade [4], respectively, with the most pronounced change occurring over the last 15 years. Arctic warming will likely continue for several decades given the current trends in global greenhouse gas emissions [5] and the lag times associated with global climate processes attaining equilibrium [6]. Hence, climate-induced effects on sea ice and polar bears will continue for several decades, or longer, if global greenhouse gas emissions are not reduced.

The ability of individuals to modify their behavior has been posited as the primary mechanism by which some animal populations have responded to climate-driven changes in their environment [7]. The best documented examples of behavioral modification in response to altered physical environments have involved changes in spatial distribution and phenological shifts (i.e., the seasonal timing of animal and plant activities, sensu [8]). For example, Perry et al. [9] documented northward shifts in distribution for a group of North Sea fishes in response to increased sea temperature. Parmesan and Yohe [10] examined over 800 terrestrial species and detected distributional shifts in approximately half: 80% of those shifts were poleward with most being influenced by the advancement of the spring season. However, species that occur at environmental extremes, such as Arctic endemics, have little opportunity to modulate climate-warming changes to their physical environment via shifts in distribution. Rather, they must display *in situ* plasticity in key behaviors or traits to cope with a changing climatic envelope.

For a habitat specialist with a long generation time such as the polar bear, the rapidly changing physical environment can create a situation where the species becomes “trapped” by its evolved response to cues that are suddenly occurring in a novel context (e.g., [11]). As a result, entrenched behaviors could become maladaptive and eventually manifest at the population level as declining vital rates—unless the species possesses sufficient phenotypic plasticity to assess and respond to highly dynamic conditions. For polar bears, there is uncertainty concerning their capacity to exhibit behavioral plasticity relative to changing sea ice phenology and availability, particularly in areas of the Arctic where bears have historically spent the entire year on the sea ice. In those areas, the decision to remain with ice as it retreats well past biologically-productive shallow waters may lead to prey scarcity and nutritional restriction [12]. By contrast, the decision to displace from retreating sea ice to shore could result in attraction to habitats that function as ecological traps because they contain inadequate resources or expose bears to novel risk factors. Understanding how polar bears respond to climate-driven displacement from primary habitat, and how this overlaps with exposure to known and novel threats, is critical to forecasting how they may fare in an increasingly dynamic environment.

Polar bears of the southern Beaufort Sea (SB) subpopulation have historically spent the entire year on the sea ice (with the exception of individuals that den on land), even when the pack ice retreated away from the coast to its minimal extent in September [1, 13]. However, over the last 15 years, the SB has experienced a marked decline in September sea ice extent, along with a pronounced lengthening of the melt season (i.e., period of time between sea ice break-up and freeze-up; [14]). The dramatic changes in the extent and phenology of sea ice habitat have coincided with evidence suggesting that use of terrestrial habitat has increased. For example, Schliebe et al. [15] estimated that between 3.7 and 8.0% of polar bears from the SB were on land in a given autumn during 2000–2005, and that percentage increased when sea ice was farthest from the coast. In contrast to the SB, polar bears of the greater Hudson Bay region [16], for example, historically spent significant periods of time on land (1–5 months) when ice completely melted each year. In general, populations in the Hudson Bay region have been demographically productive [17, 18, 19], although an increase in the length of the ice-free season has resulted in a decline in the western Hudson Bay (WH) subpopulation [20, 21] followed later by apparent stabilization [22]. In the SB, measured declines in polar bear body condition, productivity, and abundance have also been linked to declining sea ice habitat [13, 23, 24, 25, 26]. It is unknown if the decline in productivity in the SB subpopulation is linked to increased use of land or to remaining on the sea ice as it retreats away from the biologically productive water of the continental shelf.

Here, we investigated polar bears from Alaska’s SB subpopulation, where rapid environmental change may be driving a divergence in space use and foraging behaviors in the form of increased land use. Specifically, our objectives were to examine (i) the long-term trend in the use of terrestrial habitat, (ii) the influence of sea ice characteristics on the phenology of movement from sea ice to terrestrial habitats and back to ice, and (iii) the spatial distribution of bears while on shore. Last, we discuss potential cascading effects of behavioral divergence and how those effects may influence population dynamics in the SB through time.

## Materials and Methods

### Ethics Statement

This research was approved under the Marine Mammal Protection Act and Endangered Species Act with U.S. Fish and Wildlife Service (USFWS) permit number MA690038. Capture protocols were approved by the U.S. Geological Survey (USGS) Institutional Animal Care and Use Committee.

### Study Area

**Fig 1:**
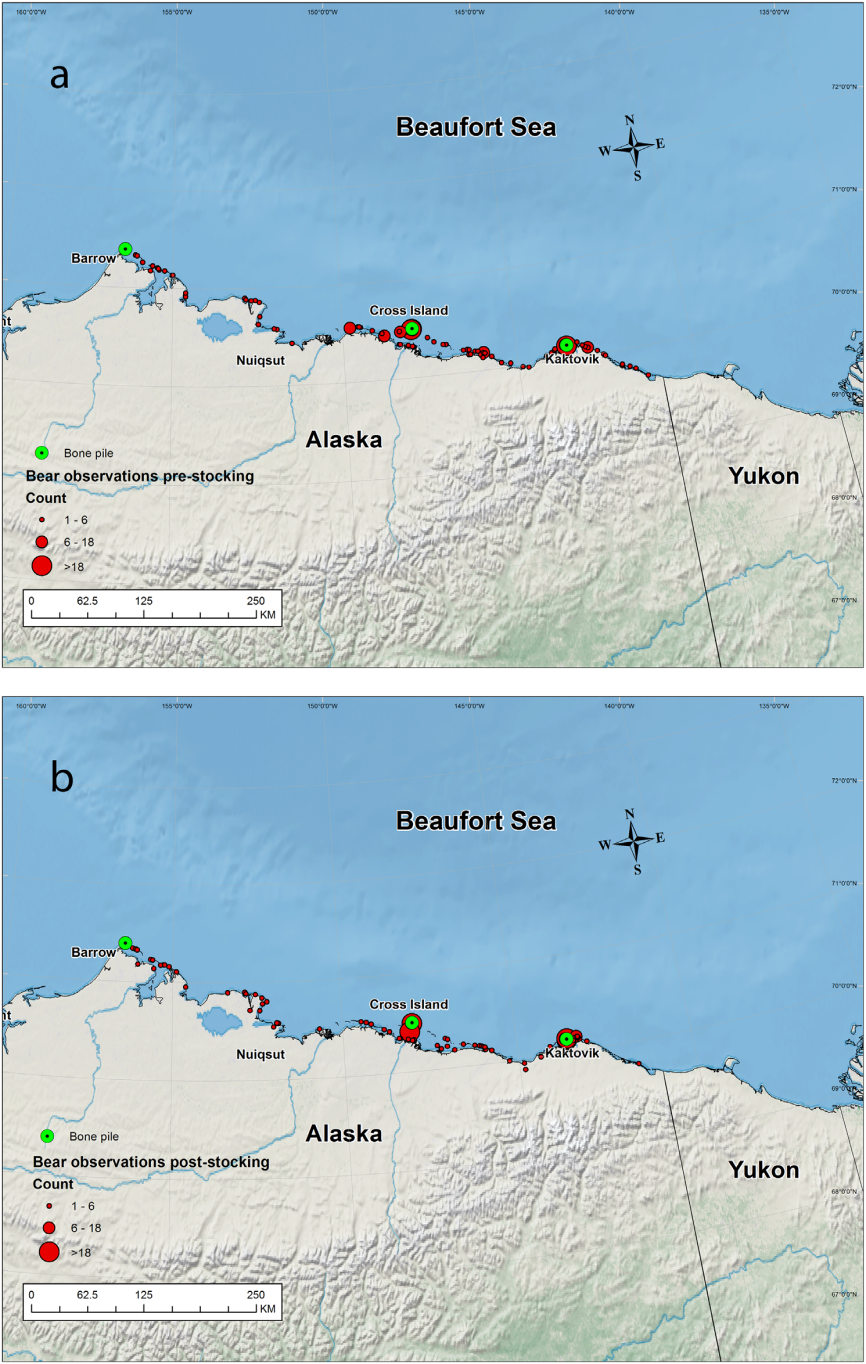
a-b. Spatial distribution of polar bears observed during fall aerial surveys, 2010–2013, along the coast and over barrier islands prior to the stocking of bowhead whale bone piles with remains from the subsistence harvest. Unused remains from subsistence-harvested bowhead whales are occasionally aggregated at sites on Point Barrow, and consistently at Cross Island (near Prudhoe Bay), and adjacent to Kaktovik on Barter Island following the cessation of the fall whaling season.

The study area ranged from Baillie Island, Canada, (70.5°N, 128° W) in the east, to Point Barrow, USA, (71°N, 156° W) in the west (Fig 1a and 1b). The SB is characterized by a narrow, biologically-productive continental shelf with bathymetry contours typically ≤ 300m, and with an abrupt shelf-break that quickly gives way to some of the deepest waters of the Arctic Ocean [27].

The SB coastal region is characterized by an industrial footprint associated with oil and gas exploration and extraction activities causing polar bears that frequent this area to be potentially exposed to industrial activities [28]. The Prudhoe Bay and Kuparuk oil fields are situated at the approximate midpoint along the coast, and the National Petroleum Reserve-Alaska (NPR-A) spans a significant stretch of the western portion of the coastal plain, though there is no significant industrial development within the NPR-A. There are 3 communities within the study area that harvest bowhead whales (*Balaena mysticetus*) in the fall: Barrow, Nuiqsut, and Kaktovik. Remains from the harvest have been sporadically aggregated at Point Barrow and consistently aggregated at Cross Island and Barter Island, all of which are nearly evenly spaced along the coast where they have served as focal attractors for polar bears [15].

### Data Collection

Polar bear research in the SB has been ongoing for over 30 years, and we used both historical and contemporary data sets to investigate whether use of land has changed over time. Since the mid-1980s, polar bears have been captured on the sea ice (up to 160 km from the coast) nearly every spring. Polar bears were encountered opportunistically from a helicopter and immobilized with the drugs sernylan or phencyclidine (prior to 1987) and tiletamine hydrochloride plus zolazepam hydrochloride (1987–2014; Telazol^®^, Fort Dodge and Warner-Lambert Co.) using a projectile syringe fired from a dart gun. A subset of adult females was fitted with either Argos or global positioning system (GPS) Platform Transmitter Terminal (PTT) satellite radio collars [13]. Age was determined by multiple methods. Cubs-of-the-year (COY) were always with their mothers and could be visually aged without error [29]. Some bears had been captured and marked in previous years, so their age was determined from their capture history. For new captures, we extracted a vestigial premolar tooth and determined age by analysis of cementum annuli [30].

### Phenology of onshore behavior

We used location data from radiocollared adult females from 1986 to 2014 to determine if bears used terrestrial habitat during summer and, if so, to generate estimates of mean date of arrival on shore, duration of time spent on shore, and mean date of departure from shore back to the sea ice. The majority of locations prior to 2010 were derived with the Argos System, and have variable levels of accuracy from < 250 m to > 1500 m (see http://www.argos-system.org/web/en/78-faq.php#faq-theme-55). We filtered locations in an attempt to remove spurious locations by first removing all designations which had a high probability of being erroneous. We then applied the Douglas Argos-Filter algorithm [31] using a maximum redundancy function set at 10 km and minimum rate (“minrate”) of movement set at 10 km/hr.

To integrate the GPS and filtered Argos location data, which varied both in accuracy and the temporal scale of collection, we employed the continuous time correlated random walk (CRAWL) model [32] to develop predicted paths at a regularized daily time interval based on observed locations. The CRAWL model allows predicted paths to take into account variable location quality and sampling intervals. Thus, for Argos locations, we defined location accuracy based on accuracy designations for Telonics Argos collars (i.e., L3: 150 m, L2: 350 m, L1: 1000 m, L0: 1500 m; http://www.telonics.com/technotes/argosintro.php). Because location accuracies are not provided for locations with LA or LB designations, we provided conservative location accuracies; LA: 5,000 m, LB: 10,000 m. We assigned locations obtained from GPS collars an accuracy of 30 m.

Based on the observed location accuracy and land use, we used the R [33] package ‘crawl’ [31] to implement the CRAWL model and predict daily polar bear locations from 1 July to 31 October period. We then associated predicted locations with buffered land coverages (described below) to determine if an animal was likely to be on land at that time. Because the CRAWL model does not provide meaningful results if observed locations are too temporally dispersed [34], we excluded predicted locations that occurred between observed points separated by >14 days. For bears that came ashore, we noted the ordinal date of arrival and departure, and calculated the total amount of time spent on shore. We then generated indices of the earliest and mean ordinal dates of arrival on shore, mean departure back to the sea ice, and length of stay on shore.

We determined if an animal’s location occurred on land by overlaying locations on one of two land coverages. The first layer was a digital elevation model (100 m resolution; http://data.eol.ucar.edu/codiac/dss/id=106.ARCSS301; accessed 12 Aug 2014) for the North Slope of Alaska. While this layer covered the majority of land used by bears in this study, it did not provide coverage for eastern Canada. Thus, to account for land use in that region, we used the default continent shapefile found in ArcGIS (version 10.1, ESRI, Redlands, CA). Because neither land coverage had sufficient resolution to detect small barrier islands, which are known to receive significant use by polar bears during summer [35], we buffered the land coverages by 5 km. We then determined which animal locations occurred within the 5 km land buffer and categorized those as predicted land locations. While this might have resulted in some bears not on land being classified as using land, this was unlikely to occur given that landfast ice is largely absent during this period. Thus, any animal observed within this buffer would most likely have used land at some point during that day.

### Sea ice characteristics

Polar bears in the SB prefer sea ice habitat over the continental shelf because it provides greater accessibility to prey than the deeper water of the polar basin [13]. We hypothesized that the phenology of land use was influenced by sea ice characteristics, including the distance between the continental shelf break and the edge of the pack ice and the concentration of ice over the shelf. We used daily sea ice data from the National Snow and Ice Data Center (NSIDC; Boulder, Colorado, USA) to develop concentration and distance metrics. Sea ice concentrations were estimated from a 25 × 25 km resolution raster of passive microwave satellite imagery [36]. For the months of July through October, we estimated a number of metrics including sea ice concentrations over the continental shelf, distances from the shelf break to pack ice, the timing of break-up and freeze-up, and length of the open-water season (see Table 1 for a list of sea ice metrics). Shelf break and shelf area were delineated by the 300 m isobath for the offshore region within the boundary of the SB polar bear subpopulation [1]. We defined areas covered by sea ice with two criteria based on different ice concentration thresholds, >15% and >50%. We then generated daily estimates of the proportion of the continental shelf area covered by >15 or 50% sea ice concentration, and the mean distance between the shelf break and the ice pack, where ice pack was comprised by ice concentrations >15 or 50%. We chose to use ice metrics based on both thresholds because >50% is most commonly cited as the threshold above which sea ice habitat is most suitable for polar bears [20], while break-up and freeze-up are often defined as the time when ≥15% concentration sea ice melts or refreezes [14]. Additionally, because the SB is characterized by a narrow continental shelf, we hypothesized that bears may remain over the productive shelf even as ice concentrations drop below 50%.

**Table 1:** Description of sea ice variables used in the analysis of factors influencing the timing of arrival on shore, length of stay, and timing of departure back to sea ice by polar bears from the Southern Beaufort Sea subpopulation.

Variable	Description
FD≤15%	The first date (day of year) when the proportion of the continental shelf covered by >15% sea ice concentration decreased to ≤15%.
FD≤50%	The first date when the proportion of the continental shelf covered by >50% sea ice concentration decreased to ≤50%.
Shelf>15%_wk	The mean proportion of the shelf covered by >15% concentration sea ice 1 week prior to arrival on shore.
Shelf>50%_wk	The mean proportion of the shelf covered by >50% concentration sea ice 1 week prior to arrival on shore.
Mdis>15%_wk	The mean distance (km) of >15% concentration sea ice from the continental shelf 1 week prior to arrival on shore.
Mdis>50%_wk	The mean distance of >50% concentration sea ice from the continental shelf 1 week prior to arrival on shore.
OW15%	The duration (number of days) of the open-water season, defined as when the proportion of the continental shelf covered by >15% sea ice concentration decreases below ≤15%.
OW50%	The duration of the open-water season, defined as when the proportion of the continental shelf covered by >50% sea ice concentration decreases below ≤50%.
Shelf>15%_OW	The mean proportion of the continental shelf covered by >15% concentration sea ice during the open water season.
Shelf>50%_OW	The mean proportion of the continental shelf covered by >50% concentration sea ice during the open water season.
Mdis>15%_OW	The mean distance of >15% concentration sea ice from the continental shelf during the open water season.
Mdis>50%_OW	The mean distance of >50% concentration sea ice from the continental shelf during the open water season.
LD≤15%	The last date when the proportion of the continental shelf covered by >15% sea ice concentration was below ≤15%.
LD≤50%	The last date when the proportion of the continental shelf covered by >50% sea ice concentration was below ≤50%.
Shelf>15%_depart	The mean proportion of the continental shelf covered by >15% concentration sea ice 1 week prior to departure from shore.
Shelf>50%_depart	The mean proportion of the continental shelf covered by >50% concentration sea ice 1 week prior to departure from shore.
Mdis>15%_depart	The mean distance of >15% concentration sea ice from the continental shelf 1 week prior to departure from shore back to sea ice.
Mdis>50%_depart	The mean distance of >50% concentration sea ice from the continental shelf 1 week prior to departure from shore back to sea ice.
Year	Calendar year in which observations were collected.

### Distribution

When polar bears of the SB come ashore, they mostly stay within a narrow band of the coast or on barrier islands [15]. From 2010 to 2013 we conducted transect-based aerial surveys twice (≤ 3 weeks apart) each fall along the coast between Point Barrow and the U.S.A.-Canada border to characterize distribution. In fall, polar bears are easy to detect when on land because of the contrast between the colors of bears and the snow- and ice-free substrate [37, 22]. Transects were 8-km in length and included segments oriented perpendicular to the coast line connected by alternating inland or coastal segments. We flew Bell 206B and Aerostar 305A helicopters at an altitude of ≈90 m and airspeed of ≈80 knots. In addition, total counts were conducted over every barrier island encountered, with the exception of Barter Island. The village of Kaktovik is located on Barter Island, and is adjacent to a bowhead whale carcass aggregation site which provides opportunities for commercial polar bear viewing. As such, we did not fly over Barter Island over concerns that helicopter activity would disturb commercial bear viewing ventures. We did, however, collect ground-based total counts of all bears present at the Barter Island carcass site and local vicinity on the same day as our aerial survey. We flew over carcass aggregation sites at Point Barrow and Cross Island, though no carcasses were present at Point Barrow in 2013. When we encountered a bear, we estimated age, sex, and group size, and collected a geographic location. We combined counts from transects and barrier islands to generate a total uncorrected minimum count for each of the two annual surveys and used the total counts to examine spatial distribution.

### Analyses

We used a generalized additive mixed model (GAMM) with a binomial distribution to determine whether the percentage of radiocollared polar bears using land ≥21 consecutive days versus remaining on the sea ice changed over time. We chose the threshold of ≥21 consecutive days because it has been used previously [35, 38] to describe long-term use of land and thus allows for comparison to our study. Based on the previously described analysis of CRAWL-derived locations, we coded land use or lack thereof by individuals as a binary response variable (i.e., 1 = individual used land, 0 = individual did not use land). Year was analyzed as a fixed effect, but because some individual bears were radiocollared in multiple years, we used individual as a random factor. We also calculated the mean annual percentage of radiocollared bears with long-term land use, and used a piecewise general linear regression procedure [39] with a normal distribution to determine if and when there was a discontinuity (i.e., breakpoint) in the mean annual percentage detected on shore over the 29 years of study. Parameters estimated for the piecewise regression included the intercept, change in slope prior to the breakpoint, and change in slope after the breakpoint [39]. We did not include collar type (Argos and GPS) as a variable in subsequent analyses, though it is possible that improvements in satellite collar technology could represent a confounding factor. However, while the ability to accurately estimate the true day of arrival on land and departure back to ice should be better with GPS-era collars, the Argos-era data should not be biased toward estimating either longer or shorter land tenures.

To determine the relationship between the phenology of onshore use by radiocollared bears and sea ice dynamics, we used linear mixed models to examine the influence of sea ice conditions and characteristics on the annual mean timing of arrival on shore, length of stay on shore, and timing of departure from shore back to the sea ice. For this analysis, we included bears that came ashore for ≥7 consecutive days and used ordinal dates of arrival and departure, and total days spent on shore as response variables. We used the ≥7 consecutive days threshold to exclude bears that used land incidentally. Because we sampled some of the same individuals repeatedly, we included individual identity as a random factor in the models with first-order autocorrelation as a covariance structure. We used restricted maximum likelihood (REML) methods for model estimation. When modeling timing of departure, we censored individuals that entered maternity dens on land. Predictor variables included measures of >15% and >50% sea ice concentrations over the continental shelf (e.g., Mn>15%, Mn>50%), distance from the shelf of >15% and >50% sea ice (Mdis>15%, Mdis>50%), and length of the open water season defined as the periods of time when sea ice concentration remained ≤15 or ≤50% (OW15%, OW50%).

We developed, *a priori*, sets of biologically plausible candidate models (S1 Table) and used Akaike’s information criterion values [40] corrected for small sample bias (AIC_c_) to aid in determining top models. We used AIC_c_ to rank and compare models based on ΔAIC_c_ and normalized Akaike weights *w*_*i*_ and considered models with ΔAIC_c_ values >2.0 to measurably differ in information content [41]. When faced with model uncertainty, we calculated 85% confidence intervals (CI) for parameter estimates to avoid unnecessarily discarding variables in models supported by lower AIC_c_ values [42]. Following Arnold [42], we considered parameters whose 85% CI overlapped zero to be uninformative. We assessed multicollinearity of predictor variables using variance inflation factors (VIF) and removed a correlated variable from a given model when VIF >10 [43]. We used normal probability plots and coefficients of correlation to ensure that model variables were normally distributed and assessed fit using measures of model deviance [44].

We used the paired sets of annual aerial surveys to investigate whether the availability of bowhead whale remains influenced polar bear distribution. We pooled data among years from surveys conducted before and after whale remains were placed at carcass aggregation sites (Point Barrow, Cross Island, and Barter Island). We used Moran’s I statistic to test the hypothesis that polar bear sightings were spatially autocorrelated (i.e., individuals were not randomly distributed) and an ArcGIS to determine the Euclidean distance of each bear sighting to the closest carcass aggregation site. We then used a Kolmogorov-Smirnov test to determine if the distribution of distances from carcass sites differed between survey sessions― i.e., whether the spatial distribution of bears differed prior to and after the stocking of carcass sites. Statistical significance for these tests was set at α = 0.05.

## Results

**Fig 2:**
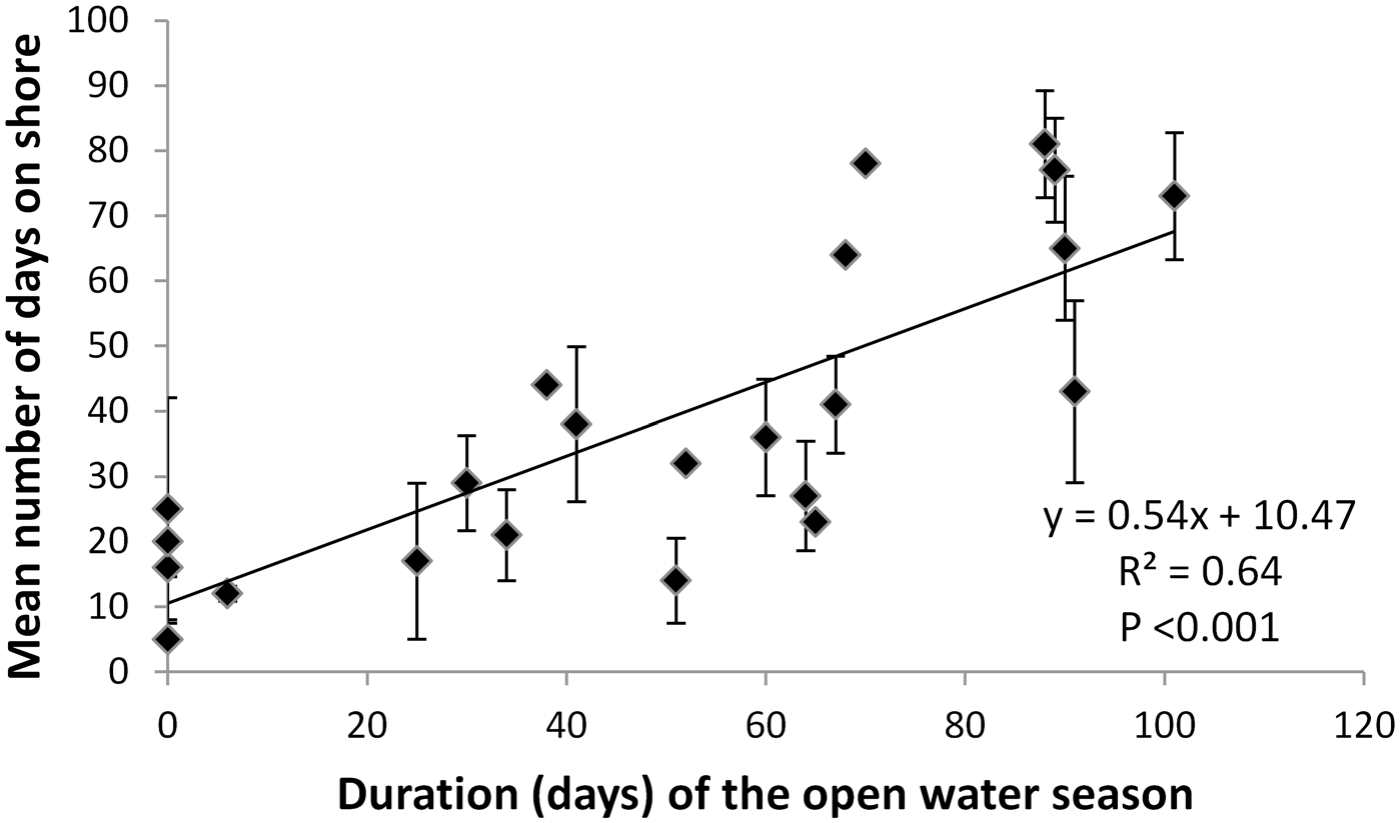
Proportion of radiocollared adult female polar bears that spent ≥ 21 consecutive days on shore, 1986–2014.

During aerial surveys conducted in fall of 2010–2013 we flew a total of 9,820 ($\overset{-}{x}$ = 1,226 ± 378 km) kilometers on transect and searched an average of 31 barrier islands to determine the spatial distribution of bears along the coast. From 1986 to 2014, a total of 389 radiocollars (satellite or GPS) were placed on 228 adult female polar bears. Results of the GAMM model indicated that the proportion of radiocollared bears coming ashore in summer and fall increased over the years (β_year_ = 0.58, P = 0.004). Using piecewise regression, we detected a breakpoint in the percentage of radiocollared bears on shore for ≥21 days at the year 2000: the average percentage of bears on shore increased from 5.8% (SE = 0.02) during 1986–1999 to 20% (SE = 0.03) during 2000–2014, reaching a high of 37% in 2013 (Fig 2).

### Onshore phenology

Among all data, 68 radiocollared (39 satellite, 29 GPS) bears representing 46 individuals spent ≥7 days shore during the open water season, which were used to characterize onshore phenology. The piecewise regression indicated that the earliest date of arrival on shore by radiocollared bears differed between the two periods ($\overset{-}{x}$_1986–1999_ = 256 (i.e., 13 September), SE = 3.9; $\overset{-}{x}$_2000–2014_ = 241 (i.e., 29 August), SE = 3.1), ranging from 6 August in 1993 to 22 July in 2000. From 1986–1999, the mean length of stay on shore was 20 days (SE = 2.5 days); from 2000–2014, the mean length of stay on shore was 56 days (SE = 3.2 days). Date of departure also varied over the years, ranging from 14 August in 1993 to 7 November in 2013 ($\overset{-}{x}$_1986–1999_ = 275 (i.e., 2 October), SE = 5.3; $\overset{-}{x}$_2000–2013_ = 294 (i.e., 21 October), SE = 1.6).

Throughout the study, polar bear arrival on shore advanced at a rate of ~5 days/decade. The top model for predicting the date of arrival of bears on shore accounted for 87% of the total model set weight. Variables contained in the top model were ordinal date when sea ice concentration over the shelf dropped below 15% (FD≤15%; β = 0.369, SE = 0.06) and the proportion of the shelf covered by >15% concentration sea ice the week prior to arrival on shore (shelf>15%_wk; β = -0.514, SE = 0.11) (Table 2). Examination of model coefficients indicated that earlier dates of <15% concentration sea ice over the shelf and increased availability of >15% concentration sea ice over the shelf resulted in earlier arrival of bears on land (Table 3). All other models for predicting the timing of arrival on shore had ΔAIC_c_ > 2 (Table 2).

**Table 2:** Model ID, explanatory variables, AIC_c_ values, Akaike weights, and deviance for linear mixed models describing the timing of arrival of polar bears on shore, 1986–2014.

Model ID	Explanatory Variables	AIC_c_	Akaike Wt. (*w*_i_)	Deviance
4	FD≤15%, Shelf>15%_wk	671.4	0.87	667.3
8	FD≤15%, Shelf>15%_wk, Mdis>15%_wk, year	676.2	0.07	672.1
7	FD≤15%, Shelf>15%_wk, Mdis>15%_wk	677.5	0.04	673.4
17	Year	686.5	0.01	682.4
1	FD≤15%	686.8	<0.00	682.7
9	FD≤50%	690.5	<0.00	686.4
6	Mdis>15%_wk, FD≤15%	692.3	<0.00	688.1
12	FD≤50%, Shelf>50%_wk	692.7	<0.00	688.6
16	FD≤50%, Shelf>50%_wk, Mdis>50%_wk, year	692.6	<0.00	688.5
14	Mdis>50%_wk, FD≤50%	697.4	<0.00	693.3
10	Shelf>50%_wk	697.9	<0.00	693.8
2	Shelf>15%_wk	699.8	<0.00	695.7
15	FD≤50%, Shelf>50%_wk, Mdis>50%_wk	699.4	<0.00	695.3
11	Mdis>50%_wk	701.2	<0.00	697.0
3	Mdis>15%_wk	702.3	<0.00	698.2
5	Mdis>15%_wk, Shelf>15%_wk	703.0	<0.00	700.6
13	Mdis>50%_wk, Shelf>50%_wk	704.7	<0.00	698.9

**Table 3:** Response and explanatory variables, model rank, AIC_c_ value, coefficient estimates, and 85% confidence intervals (CI) for the top general linear models describing the phenology of land use, 1986–2014.

Response	Model ID	Explanatory Variables	Mode Rank	Estimate (β)	S.E.	85% CI lower	85% CI upper	P-value
Arrival date	4	FD≤15%	1	0.369	0.06	0.27	0.45	<0.0001
		Shelf>15%_wk		-0.515	0.11	-0.68	-0.35	<0.0001
Length on shore	7	OW15%	1	0.334	0.11	0.17	-0.01	0.002
		Mdis>15%_OW		0.022	0.02	0.01	0.07	0.22
		Year		0.907	0.48	0.19	1.62	0.06
Departure date	16	Shelf>15%_depart	1	-0.158	0.11	-0.32	0.01	0.005
		Mdis>15%_depart		-0.118	0.02	-0.15	-0.09	0.21
		Year		1.059	0.36	0.52	1.59	0.03

**Fig 3:**
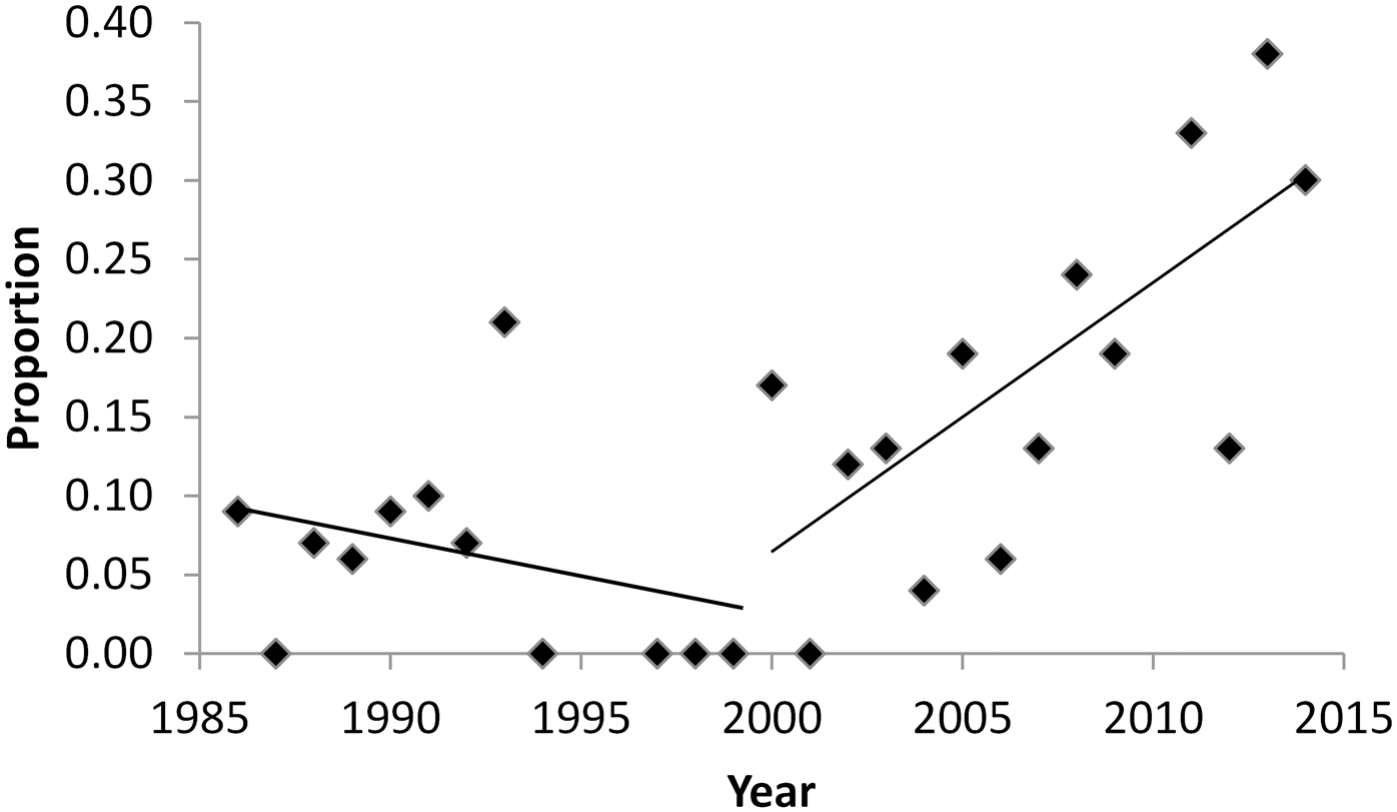
Mean (and standard error) length of stay on shore relative to the length of the open water season, defined as when the proportion of the continental shelf covered by >15% sea ice concentration decreases below ≤15%.

Over the course of the study, the total days spent on shore by polar bears increased by ~7 days/decade. The top model for predicting total days spent on shore by polar bears accounted for 74% of the model set weight and contained the mean distance of >15% concentration sea ice from the continental shelf during the open water season (Mdis>15%_OW; β = 0.022, SE = 0.02), duration of the open water season (defined using the 15% threshold; OW15%; β = 0.334, SE = 0.11), and year (β = 0.907, SE = 0.48) (Table 4). Examination of model coefficients indicated that total number of days spent on shore increased with increasing distance of >15% sea ice from the shelf, duration of the open water season (F_61,14_ = 8.90, P < 0.0001; Fig 3), and year. However, the 85% confidence interval for Mdis>15%_OW overlapped zero, indicating the variable may be uninformative [42]. All other models for predicting the length of stay on shore had ΔAIC_c_ >2 (Table 4).

**Table 4:** Model ID, explanatory variables, AIC and AIC_c_ values, Akaike weights, and deviance for the linear mixed models describing the length of stay on shore, 1986–2014.

Model ID	Explanatory Variables	AIC_c_	Akaike Wt. (*w*_i_)	Deviance
7	Mdis>15%_OW, OW15%, year	658.7	0.74	656.6
1	OW15%	662.5	0.11	658.3
15	Year	663.8	0.06	659.7
4	Mdis>15%_OW, OW15%	664.6	0.04	660.4
13	Shelf>50_OW, Mdis>50%_OW, year	666.0	0.02	663.9
14	OW50%, Mdis>50_OW, year	666.2	0.01	662.0
6	Shelf>15_OW, Mdis>15_OW, year	666.8	0.01	664.8
11	Mdis>50_OW, OW50%	673.5	<0.00	669.3
8	OW50%	679.6	<0.00	675.4
12	Shelf>50_OW, Mdis>50_OW	680.6	<0.00	678.6
5	Shelf>15_OW, Mdis>15_OW	681.4	<0.00	679.3
10	Mdis>50_OW	682.1	<0.00	678.0
2	Shelf>15_OW	683.2	<0.00	679.0
3	Mdis>15_OW	686.2	<0.00	682.1
9	Shelf>50_OW	687.7	<0.00	683.6

The top model for predicting the timing of departure of bears from shore back to the sea ice explained 77% of the model set weight and contained the proportion of the shelf covered by >15% concentration sea ice the week prior to departure (Shelf>15%_depart; β = -0.158, SE = 0.11), the mean distance of >15% sea ice concentration from the shelf (Mdis>15%_depart; β = -0.118, SE = 0.02), and year (β = 1.059, SE = 0.26) (Table 5). The 85% confidence interval for the proportion of the shelf covered by >15% concentration sea ice the week prior to departure overlapped zero, suggesting it may be an uninformative variable. All other models for predicting the timing of departure from shore back to sea ice had ΔAIC_c_ >2 (Table 5). Inspection of model coefficients indicated that decreased availability of >15% concentration sea ice, reduced distance of >15% sea ice from the shelf, and later year resulted in later departure of bears from shore back to sea ice. Comparison of *w*_*i*_ for the first- and second-ranked models indicated that the first-ranked model was 4.5 times more likely to be the actual best model and deviance statistics indicated the top model best fit the data (Table 5). Over the duration of the study, departure from shore back to sea ice occurred approximately 7 days later/decade.

**Table 5:** Model ID, explanatory variables, AIC and AIC_c_ values, Akaike weights, and deviance for the linear mixed models describing the timing of departure from shore back to sea ice, 1986–2014.

Model ID	Explanatory Variables	AIC_c_	Akaike Wt. (*w*_i_)	Deviance
7	Shelf>15%_depart, Mdis>15%_depart, year	527.2	0.77	525.1
16	Shelf>50%_depart, Mdis>50%_depart, year	530.2	0.17	528.1
13	LD≤50%, Mdis>50%_depart	534.4	0.02	532.3
6	Shelf>15%_depart, Mdis>15%_depart	535.1	0.01	533.0
15	LD≤50%, Shelf>50%_depart, Mdis>50%_depart	535.7	0.01	533.6
14	Shelf>50%_depart, Mdis>50%_depart	537.1	<0.00	535.0
8	LD≤15%, Shelf>15%_depart, Mdis>15%_depart	529.2	<0.00	537.1
5	LD≤15%, Mdis>15%_depart	540.6	<0.00	538.5
17	year	540.6	<0.00	538.6
2	Mdis>15%_depart	543.5	<0.00	539.4
10	Mdis>50%_depart	544.7	<0.00	540.4
9	LD≤50%	544.9	<0.00	542.8
12	LD≤50%, Shelf>50%_depart	546.4	<0.00	544.3
11	Shelf>50%_depart	547.8	<0.00	545.7
3	Shelf>15%_depart	549.0	<0.00	546.9
1	LD≤15%	550.0	<0.00	547.9
4	LD≤15%, Shelf>15%_depart	551.6	<0.00	549.5

### Distribution on land

Moran’s I statistic indicated that polar bears were not randomly distributed when observed during aerial surveys conducted prior to (z = 8.51, P <0.0001; Fig 1a) and after (z = 15.08, P < 0.0001; Fig 1b) the stocking of bowhead whale remains sites in 2010–2013. The percentage of polar bears located in close proximity to bowhead whale remains sites was greater following stocking (D = 0.14, P = 0.001). Prior to stocking, 64% of polar bear observations occurred within 16 km (i.e., mean daily distance traveled by SB polar bears; [45]) of a site. After stocking 78% of all bears observed were within 16 km of a site. During surveys conducted before and after bowhead whales were harvested, we observed the greatest percentage of bears near Barter Island (40%), followed by Cross Island (33%). Relatively few bears were observed in the vicinity of Point Barrow (<2%).

## Discussion

Historical (i.e., pre-2000) use of terrestrial habitat during the open-water season by SB polar bears was relatively rare and limited to short durations [45]. Recently, land-use behavior has become more prevalent, although a majority of the SB subpopulation still remains on the sea ice during summer. We detected clear trends over time of 1) an increasing percentage of polar bears coming ashore (the percentage tripled from 2000–2014), 2) earlier dates of arrival (advancing at a rate of ~ 5 days/decade), 3) later dates of departure (~7 days later/decade), and 4) longer tenure on land (increased at a rate of ~7 days/decade). Further, increased use of terrestrial habitat was related to declines in sea ice extent and changes in sea ice phenology. Since the late 1990s, the duration of the open-water season in the SB increased by an average of 32 and 36 days based on >50% and >15% sea ice concentrations over the continental shelf, respectively, while the amount of time spent on land increased by ~3 weeks. Our results are consistent with other recent work showing increased land use by polar bears from the adjacent Chukchi Sea subpopulation over roughly the same time period [38].

The relatively infrequent historical use of land by SB polar bears was likely due to the persistent availability of sea ice over the continental shelf, even during the period of minimum sea ice extent in September. Since the late 1990s, the duration of the open-water season in the SB increased by an average of 66% or 82% (depending on sea ice concentration threshold), while the September average distance from shore to pack ice increased by 120%. Since the 2000s, the length of the open-water season has increased at a rate of ≈9 days/decade, which is among the largest rates of increase for the seas of the Arctic Ocean [14]. From 2006 to 2014, the distance from shore to September pack ice has increased an additional 65%, which placed the leading edge of the ice an average of 450 km from the continental shelf. Polar bears prefer to forage from sea ice over shallow, biologically productive continental shelf waters [13]. The lengthening period of sea ice absence over the shelf during summer equates to an increasing loss of preferred foraging habitat. Evidence suggests that displaced polar bears are increasingly coming ashore in response to this loss of sea ice habitat.

Previous work in the SB [15] and elsewhere (e.g., WH; [20]) has found that the timing of arrival of bears on shore was associated with sea ice dropping below a 50% concentration. More recently, Cherry et al. [46] evaluated multiple sea ice concentration thresholds in WH and determined that dates of arrival were best correlated with the timing of 30% sea ice concentration, while departure occurred after ice concentrations reached >10%. Our findings, that the availability of sea ice concentrations >15% (but <50%) are best correlated with the timing of arrival, length of stay, and timing of departure of SB bears, is qualitatively similar to the findings of Cherry et al. [46]. It appears that in both subpopulations, polar bears delay the transition from ice to shore until ice drops below a concentration where its use as a reliable substrate is untenable. Interestingly, our finding of an inverse relationship between timing of arrival and concentration of >15% ice over the shelf suggests that bears may come ashore before widespread disappearance of low concentration ice in order to avoid long-distance swims [47]. Collectively, our findings provide important quantitative evidence of the relationship between sea ice phenology and use of terrestrial habitat by polar bears. Monitoring the timing and rate of seasonal ice disappearance may be an effective, logistically tractable way for managers and industry to prepare for the annual arrival of bears on shore.

We found a notable increase in the proportion of radiocollared bears coming ashore in summer and fall beginning in the year 2000. From 2004 to 2007, there was a pronounced decline in the survival of SB polar bears, followed by two years (2008–2009) of apparent stability [26]. The declines and subsequent stability of survival and abundance occurred as use of terrestrial habitat was increasing. While there is no causal link between the patterns in polar bear vital rates and increased use of terrestrial habitat, there is precedence in other species for behavioral shifts ameliorating some of the adverse effects of rapid environmental change. For example, Charmentier et al. [48] found that individual adjustment of behavior allowed a population of great tits (*Parus major*) to closely track changes in prey phenology and maintain the temporal match between clutch hatch date and peak availability of prey. This suggests that behavioral adjustments that closely track key phenological shifts may lessen some impacts of rapid environmental change, at least in the short term. The decision by some polar bears from the SB to exploit terrestrial habitat, rather than remain with the retreating pack ice, appears to be a behavioral response to the loss of sea ice habitat over the continental shelf. This behavior is not necessarily surprising since other subpopulations where the sea ice completely melts every summer (e.g., WH, southern Hudson Bay, Foxe Basin, and Davis Strait) display greater use of land along with flexibility in foraging behavior [49]. In the near-term, whether bears benefit from this behavioral flexibility will likely hinge on the trade-off between the availability of food resources (and net energetic benefit), and the risks associated with accessing them, such as increased exposure to human-related activities, competition with grizzly bears (*Ursus arctos*) [50], and increased potential for disease transmission [51]. However, for polar bears to benefit over the long term, behavioral flexibility will have to result in adaptations to environmental change on a sufficiently fast time-scale to result in evolutionary rescue [52].

Distribution data obtained from aerial surveys suggests that bowhead whale bone piles are focal attractors for bears on shore. Rogers et al. [53] found evidence of a shift in foraging behavior by some SB polar bears marked by fidelity to the nearshore region in winter and spring and consumption of bowhead whale tissue during summer and fall. It is likely that most bowhead whale tissue is consumed by bears visiting sites that have been stocked with remains following fall whaling [54], though scavenging on beach-cast whales also occurs. Nevertheless, the difference in the biomass of marine mammal food resources available to bears on shore is an important distinction between the SB and the previously mentioned five subpopulations of polar bears that have historically used land in summer. For the latter, entire subpopulations come ashore when the annual ice melts completely each summer and bears enter a hypophagic state until the ice reforms in the fall [1, 55, 56]. In WH, the open water season lasts upwards of 4 months (e.g., [57]) and model-based estimates, that assume polar bears fast while on shore, suggest that an increase beyond 5 months could trigger substantial declines in reproductive potential and survival ([58, 59, 60] but see [61]). Currently in the SB, bears are spending upwards of 2.5 months on shore and usually have access to bowhead whale remains for the latter portion of that period. If the trends of increasing use of terrestrial habitat and lengthening open water season continue in the SB, then any relative benefits of scavenging bowhead whale remains should diminish over time (assuming biomass available to bears remains consistent).

Increased use of terrestrial habitat and exploitation of human-provisioned resources by polar bears has attendant risks, including a greater potential for human-polar bear interaction and conflict. Wildlife-human conflict can have wide-ranging effects, including adversely impacting wildlife populations, causing economic losses to stakeholders, and endangering public safety [62]. The north coast of Alaska includes several villages and an industrial footprint associated with oil exploration and extraction activities, all of which are in relatively close proximity to bowhead whale remains sites (particularly at Barter and Cross Islands) where the majority of bears were detected during aerial surveys. Human-wildlife conflicts are often clustered in space and time (e.g., [63]) due to the availability and distribution of focal attractors. Given that the extent of summer sea ice is projected to decline through the 21^st^ century [64], terrestrial habitat and human-provisioned resources are likely to become increasingly important for SB polar bears. Bears that are highly motivated to obtain food appear more willing to risk interacting with humans (e.g., [65]), and the increased frequency of bears on land, coupled with expanding human activity due to retreating sea ice, is expected to lead to greater human-polar bear interaction and conflict. Proactive management of human-polar bear interactions will be needed to reduce the future risk of conflict.

Our study suggests that SB polar bears have become more reliant on terrestrial habitat. Since the mid-2000s, the estimated proportion of the SB subpopulation coming ashore [15] has increased substantially and the behavior should no longer be considered trivial, even though the majority of the subpopulation still remains with the sea ice during the open-water season. Indeed, there is reason to hypothesize that use of terrestrial habitat may be adaptive, at least for the short-term. When summer sea ice persists in the SB, it is now relegated to the deep water of the polar basin which is less biologically productive than the continental shelf region. As a result, polar bears that remain with the ice may have fewer opportunities to encounter ringed (*Pusa hispida*) and bearded seals (*Erignathus barbatus*), which may explain reports of increased frequency of fasting, decreased kill rates [66, 67], and declining body condition [24]. By contrast, polar bears that come ashore and scavenge bowhead whales may be able to maximize energy intake while minimizing energy expended, thereby reducing the likelihood of fasting and staving off declines in body condition.

Polar bears have evolved preferences for sea ice habitat and preying on marine mammals. In the SB, those preferences are informing two seemingly disparate strategies for coping with the loss of summer sea ice habitat: displace to shore and scavenge on predictably-available marine mammal food, or remain with the sea ice as it retracts over the polar basin and risk nutritional restriction [12]. Human-induced rapid environmental change is having profound effects on the quality and quantity of Arctic sea ice [68, 69], which will likely make it difficult for polar bears and other ice-adapted species to reliably select suitable habitats for maintaining fitness [70]. Behavioral plasticity is the initial response to dramatic environmental perturbations, followed by transmission of innovative behaviors within and across generations, eventually leading to evolution of the behavioral response over time [71] and, perhaps, evolutionary rescue [52]. However, behavioral plasticity may be an effective response by polar bears only if the rate of environmental change does not outpace transmission of behavioral innovations.

## Supporting Information

S1 Table Hypotheses and candidate linear regression models tested to predict the timing of arrival on shore, length of stay on shore, and timing of departure from shore by adult female polar bears, 1986–2014. (DOCX)
