# Supplementary material for: Rapid Environmental Change Drives Increased Land Use by an Arctic Marine Predator
Source: PLoS One. 2016 Jun 1;11(6):e0155932. doi: 10.1371/journal.pone.0155932 (PMC4889047; doi:10.1371/journal.pone.0155932)
Supplement: S1 Table — (DOCX) [file pone.0155932.s001.docx]

S1 Table. Hypotheses and candidate linear regression models tested to predict the timing of arrival on shore, length of stay on shore, and timing of departure from shore by adult female polar bears, 1986-2014.

| **Model Set** | **Model ID** | **Hypothesis description** | **Model variables** |
| --- | --- | --- | --- |
| Timing of arrival | 1 | Arrival is influenced by the first date when the percentage of the continental shelf covered by >15% sea ice concentration decreased to ≤15%. | FD≤15% |
|  | 2 | Arrival is influenced by the mean percentage of the continental shelf covered by >15% concentration sea ice 1 week prior to arrival on shore. | Shelf>15%_wk |
|  | 3 | Arrival is influenced by the mean distance of >15% concentration sea ice from the continental shelf 1 week prior to arrival on shore. | Mdis>15%_wk |
|  | 4 | Arrival is influenced by the first date when the percentage of the continental shelf covered by >15% sea ice concentration decreased to ≤15%, and the mean percentage of the shelf covered by >15% concentration sea ice 1 week prior to arrival on shore. | FD≤15%  Shelf>15%_wk |
|  | 5 | Arrival is influenced by the mean distance of >15% concentration sea ice from the continental shelf 1 week prior to arrival on shore, and the mean percentage of the shelf covered by >15% concentration sea ice 1 week prior to arrival on shore. | Mdis>15%_wk  Shelf>15%_wk |
|  | 6 | Arrival is influenced by the mean distance of >15% concentration sea ice from the continental shelf 1 week prior to arrival on shore, and the first date when the percentage of the shelf covered by >15% sea ice concentration decreased to ≤15%. | Mdis>15%_wk  FD≤15% |
|  | 7 | Arrival is influenced by the first date when the percentage of the continental shelf covered by >15% sea ice concentration decreased to ≤15%, the mean percentage of the shelf covered by >15% concentration sea ice 1 week prior to arrival, and the mean distance of >15% concentration sea ice from the shelf 1 week prior to arrival. | FD≤15%  Shelf>15%_wk  Mdis>15%_wk |
|  | 8 | Arrival is influenced by the first date when the percentage of the continental shelf covered by >15% sea ice concentration decreased to ≤15%, the mean percentage of the shelf covered by >15% concentration sea ice 1 week prior to arrival, the mean distance of >15% concentration sea ice from the shelf 1 week prior to arrival, and year. | FD≤15%  Shelf>15%_wk  Mdis>15%_wk  Year |
|  | 9 | Arrival is influenced by the first date when the percentage of the continental shelf covered by >50% sea ice concentration decreased to ≤50%. | FD≤50% |
|  | 10 | Arrival is influenced by the mean percentage of the continental shelf covered by >50% concentration sea ice 1 week prior to arrival on shore. | Shelf>50%_wk |
|  | 11 | Arrival is influenced by the mean distance of >50% concentration sea ice from the continental shelf 1 week prior to arrival on shore. | Mdis>50%_wk |
|  | 12 | Arrival is influenced by the first date when the percentage of the continental shelf covered by >50% sea ice concentration decreased to ≤50%, and the mean percentage of the shelf covered by >50% concentration sea ice 1 week prior to arrival. | FD≤50%  Shelf>50%_wk |
|  | 13 | Arrival is influenced by the mean distance of >50% concentration sea ice from the continental shelf 1 week prior to arrival, and the mean percentage of the shelf covered by >50% concentration sea ice 1 week prior to arrival. | Mdis>50%_wk  Shelf>50%_wk |
|  | 14 | Arrival is influenced by the mean distance of >50% concentration sea ice from the continental shelf 1 week prior to arrival, and the first date when the percentage of the shelf covered by >50% sea ice concentration decreased to ≤50%. | Mdis>50%_wk  FD≤50% |
|  | 15 | Arrival is influenced by the first date when the percentage of the continental shelf covered by >50% sea ice concentration decreased to ≤50%, the mean percentage of the shelf covered by >50% concentration sea ice 1 week prior to arrival, and the mean distance of >50% concentration sea ice from the shelf 1 week prior to arrival. | FD≤50%  Shelf>50%_wk  Mdis>50%_wk |
|  | 16 | Arrival is influenced by the first date when the percentage of the continental shelf covered by >50% sea ice concentration decreased to ≤50%, the mean percentage of the shelf covered by >50% concentration sea ice 1 week prior to arrival, the mean distance of >50% concentration sea ice from the shelf 1 week prior to arrival, and year. | FD≤50%  Shelf>50%_wk  Mdis>50%_wk  Year |
|  | 17 | Arrival varies by year. | Year |
|  |  |  |  |
| Length of stay | 1 | Length of stay on shore is influenced by the duration of the open-water season, defined as the period of time when the percentage of the continental shelf covered by >15% sea ice concentration is ≤15%. | OW15% |
|  | 2 | Length of stay on shore is influenced by the percentage of the shelf covered by >15% concentration sea ice during the open water season. | Shelf>15%_OW |
|  | 3 | Length of stay on shore is influenced by the mean distance of >15% concentration sea ice from the continental shelf during the open water season. | Mdis>15%_OW |
|  | 4 | Length of stay on shore is influenced by the mean distance of >15% sea ice concentration from shore and the duration of the 15% open-water season. | Mdis>15%_OW  OW15% |
|  | 5 | Length of stay on shore is influenced by the percentage of the shelf covered by >15% concentration sea ice and the mean distance of >15% concentration sea ice from the shelf during the open water season. | Shelf>15%_OW  Mdis>15%_OW |
|  | 6 | Length of stay on shore is influenced by the percentage of the shelf covered by >15% concentration sea, the mean distance of >15% concentration sea ice from the shelf, and year. | Shelf>15%_OW  Mdis>15%_OW  Year |
|  | 7 | Length of stay on shore is influenced by the duration of the 15% open water season, the mean distance of >15% concentration sea ice from the shelf, and year. | OW15%  Mdis>15%_OW  Year |
|  | 8 | Length of stay on shore is influenced by the duration of the open-water season, defined as the period of time when the percentage of the continental shelf covered by >50% sea ice concentration is ≤50%. | OW50% |
|  | 9 | Length of stay on shore is influenced by the perceentage of the shelf covered by >50% concentration sea ice during the open water season. | Shelf>50%_OW |
|  | 10 | Length of stay on shore is influenced by the mean distance of >50% concentration sea ice from the continental shelf during the open water season. | Mdis>50%_OW |
|  | 11 | Length of stay on shore is influenced by the mean distance of >50% sea ice concentration from shore and the duration of the 50% open-water season. | Mdis>50%_OW  OW50% |
|  | 12 | Length of stay on shore is influenced by the percentage of the shelf covered by >50% concentration sea ice and the mean distance of >50% concentration sea ice from the shelf during the open water season. | Shelf>50%_OW  Mdis>50%_OW |
|  | 13 | Length of stay on shore is influenced by the percentage of the shelf covered by >50% concentration sea, the mean distance of >50% concentration sea ice from the shelf, and year. | Shelf>50%_OW  Mdis>50%_OW  Year |
|  | 14 | Length of stay on shore is influenced by the duration of the 50% open water season, the mean distance of >50% concentration sea ice from the shelf, and year. | OW50%  Mdis>50%_OW  Year |
|  | 15 | Length of stay varies by year. | Year |
|  |  |  |  |
| Timing of departure | 1 | Departure is influenced by the last date when the percentage of the continental shelf covered by 15% sea ice concentration was below 15%. | LD<15% |
|  | 2 | Departure is influenced by the mean distance of >15% sea ice concentration from the shelf 1 week prior to departure. | Mdis>15%_depart |
|  | 3 | Departure is influenced by the percentage of the shelf covered by >15% concentration ice 1 week prior to departure. | Shelf>15%_depart |
|  | 4 | Departure is influenced by the last date when the percentage of the continental shelf covered by >15% sea ice concentration was below 15%, and the percentage of the shelf covered by >15% concentration ice 1 week prior to departure. | LD<15%  Shelf>15%_depart |
|  | 5 | Departure is influenced by the last date when the percentage of the continental shelf covered by >15% sea ice concentration was below 15%, and the mean distance of >15% sea ice concentration from the shelf 1 week prior to departure. | LD<15%  Mdis>15%_depart |
|  | 6 | Departure is influenced by the mean distance of >15% sea ice concentration from the shelf 1 week prior to departure, and the percentage of the shelf covered by >15% concentration ice 1 week prior to departure. | Mdis>15%_depart  Shelf>15%_depart |
|  | 7 | Departure is influenced by the last date when the percentage of the continental shelf covered by >15% sea ice concentration was below 15%, the percentage of the shelf covered by >15% concentration ice 1 week prior to departure, and the mean distance of >15% sea ice concentration from the shelf 1 week prior to departure. | LD<15%  Shelf>15%_depart  Mdis>15%_depart |
|  | 8 | Departure is influenced by the last date when the percentage of the continental shelf covered by >15% sea ice concentration was below 15%, the percentage of the shelf covered by >15% concentration ice 1 week prior to departure, the mean distance of >15% sea ice concentration from the shelf 1 week prior to departure, and year. | LD<15%  Shelf>15%_depart  Mdis>15%_depart  Year |
|  | 9 | Departure is influenced by the last date when the percentage of the continental shelf covered by 50% sea ice concentration was below 50%. | LD<50% |
|  | 10 | Departure is influenced by the mean distance of >50% sea ice concentration from the shelf 1 week prior to departure. | Mdis>50%_depart |
|  | 11 | Departure is influenced by the percentage of the shelf covered by >50% concentration ice 1 week prior to departure. | Shelf>50%_depart |
|  | 12 | Departure is influenced by the last date when the percentage of the continental shelf covered by >50% sea ice concentration was below 50%, and the percentage of the shelf covered by >50% concentration ice 1 week prior to departure. | LD<50%  Shelf>50%_depart |
|  | 13 | Departure is influenced by the last date when the percentage of the continental shelf covered by >50% sea ice concentration was below 50%, and the mean distance of >50% sea ice concentration from the shelf 1 week prior to departure. | LD<50%  Mdis>50%_depart |
|  | 14 | Departure is influenced by the mean distance of >50% sea ice concentration from the shelf 1 week prior to departure, and the percentage of the shelf covered by >50% concentration ice 1 week prior to departure. | Mdis>50%_depart  Shelf>50%_depart |
|  | 15 | Departure is influenced by the last date when the percentage of the continental shelf covered by >50% sea ice concentration was below 50%, the ercentage of the shelf covered by >50% concentration ice 1 week prior to departure, and the mean distance of >50% sea ice concentration from the shelf 1 week prior to departure. | LD<50%  Shelf>50%_depart  Mdis>50%_depart |
|  | 16 | Departure is influenced by the last date when the percentage of the continental shelf covered by >50% sea ice concentration was below 50%, the percentage of the shelf covered by >50% concentration ice 1 week prior to departure, the mean distance of >50% sea ice concentration from the shelf 1 week prior to departure, and year. | LD<50%  Shelf>50%_depart  Mdis>50%_depart  Year |
|  | 17 | Timing of departure varies by year. | Year |
